# Growth Promoting Effect of Hyaluronan Synthesis Promoting Substances on Japanese Eel Leptocephali

**DOI:** 10.1371/journal.pone.0098688

**Published:** 2014-06-04

**Authors:** Yutaka Kawakami, Kazuharu Nomura, Hideki Tanaka

**Affiliations:** Nansei Station, National Research Institute of Aquaculture, Fisheries Research Agency, Minamiise, Japan; Casey Eye Institute, United States of America

## Abstract

Hyaluronans (HAs) are glycosaminoglycans produced in the bodies of Anguilliform and Elopiform leptocephali, and play a role in metabolic energy. In mammals, HA synthesis-promoting substances (HASPS) up-regulate the expression of HA synthase (HAS) and increase the amount of HA in the body. In this study, Japanese eel leptocephali were fed a HASPS containing diet. We analyzed HAS1s and HAS2 expression, HA content, and their influence on growth. HASPS extracted from *Grifola frondosa* promoted HAS1s and HAS2 mRNA and HA content. Other than mammals, these results are first reported in vertebrate. Moreover, HASPS extracted from *G. frondosa* promoted leptocephalus growth. The relationship between growth and HA in the leptocephali is not yet clear. However, based on our results we hypothesize that HA is involved in the storage of energy, which is metabolized to sugars when needed for metabolic energy.

## Introduction

The Japanese eel (*Anguilla japonica*) is an important commercial species in Japan owing to its high market value as a food source. In 2010, successful closed-cycle breeding of the Japanese eel was reported [1]. However, production of artificial seeding for industry has not been established. To culture Japanese eel, 100% natural glass-eels, which migrate to the Japanese coast and collected in the rivers, are used for seeding. Closed-cycle breeding fish show improved growth and easier breeding compared with wild fish. Kawakami et al. [2], [3] reported that wild Japanese eel larvae, leptocephali, start metamorphosing at 4- to 5-months-old using otolith daily increment analysis. In contrast, cultured leptocephalus metamorphosis begins at more than 200 days post hatching (dph) [4], [5]. The average duration from hatched larvae to glass-eel is about 299 days (minimum to maximum: 153–754 days) [6]; this is longer in cultured than in wild leptocephali. Low growth rate is considered to be one of the reasons for this phenomenon. In the ocean, Japanese leptocephali feed on readily available particulate material originating from various sources closely linked to ocean primary production [7]. The artificial diet for cultured leptocephali is based on shark eggs [1], [4]. The present breeding system, including the artificial diet, does not reflect the eel's natural environment. The annual production of glass-eel in recent years has been less than 1,000 individuals in Japan [6]. For large-scale glass-eel production, shortening of the breeding duration is desirable; to do this, development of a new breeding system and/or upgrading the present breeding system is necessary.

Hyaluronan (hyaluronic acid, HA), a high-molecular-weight linear glycosaminoglycan (GAG) consisting of alternating glucuronic acid (GlcUA) and *N*-acetylglucosamine (GlcNAc) residues, is a major component of most extracellular matrices [8], [9]. Accumulation of HA is correlated with cell proliferation and migration in several developing tissues and organs [10], [11]. Moreover, HA plays a role in tissue water homeostasis [9]. Anguilliform and Elopiform leptocephali produce GAGs; most of which are HAs [12]. In the Japanese conger eel (*Conger myriaster*), about 50% of its dry body weight is HA, which degrades with body water content during metamorphosis [13]. It is notable that in bonefish (*Albula* sp.) leptocephali some metabolic energy is provided by GAGs during metamorphosis [14]. In short, it may be possible that HA in Japanese eel leptocephali also plays a role in storing polysaccharides as glycogen. Furthermore, by enhancing HA synthesis, it may be possible to enhance Japanese eel leptocephalus growth.

In a previous study, *Grifola frondosa* extract enhanced hyaluronan synthetase (HAS) and HA in human cutaneous fibroblasts *in vitro* [15]. In addition, some seaweed extracts also enhanced HAS and HA in rat cutaneous primary cells in culture [16]; however, it is not known if these extracts enhance HA synthesis in teleosts. The aim of this study is to elucidate whether or not administration of *G. frondosa* extract by feeding enhances HA synthesis and influences the growth associated with it in Japanese eel leptocephali. First, we cloned Japanese eel HAS genes and analyzed their function. Second, we estimated hyaluronan synthesis enhancement by *G. frondosa* extract by feeding the extract to first feeding larvae and investigated HAS gene expression patterns. Finally, we assessed the influence of HA accumulation on larval growth through long term feeding experiments with *G. frondosa* extract.

## Results and Discussion

**Figure 1:**
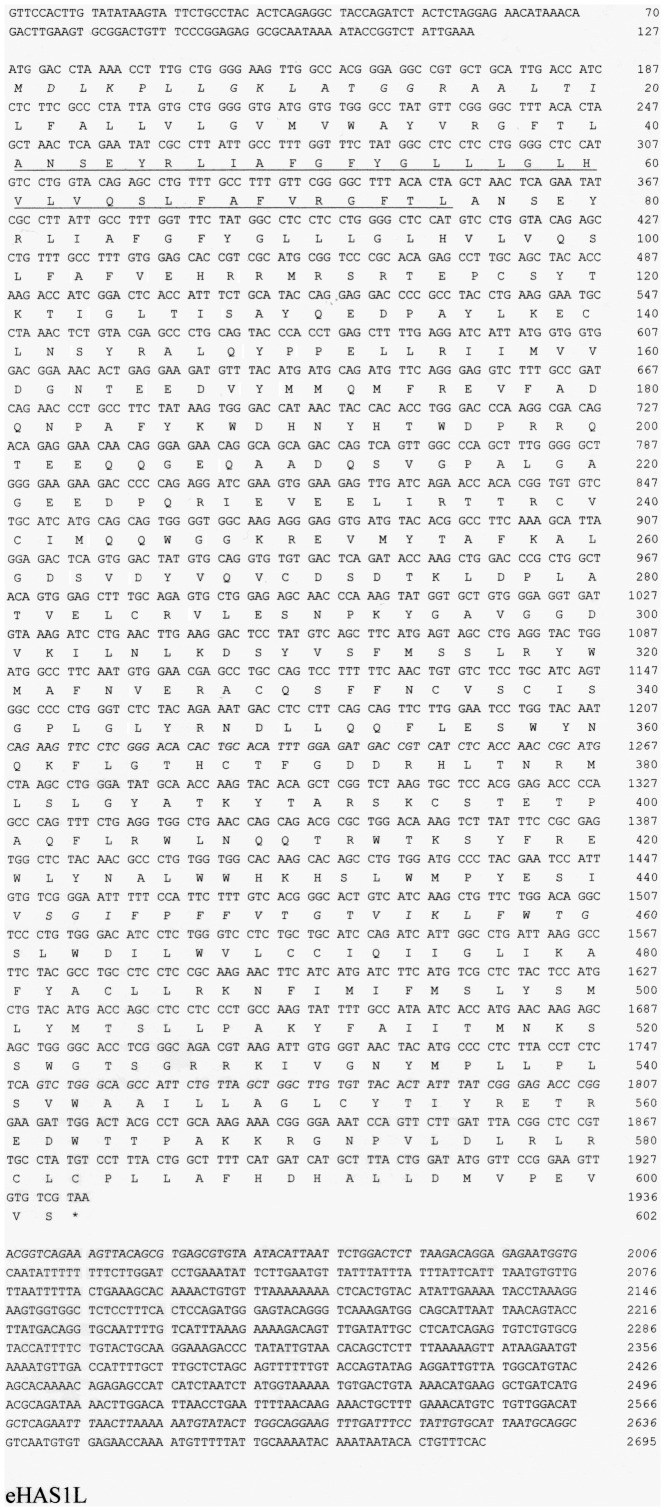
Nucleotide sequence of Japanese eel hyaluronan synthase 1s (eHAS1s) with the predicted amino acid sequence. The insertion in the splice variant of eHAS1 (eHAS1L) is underlined.

**Figure 2:**
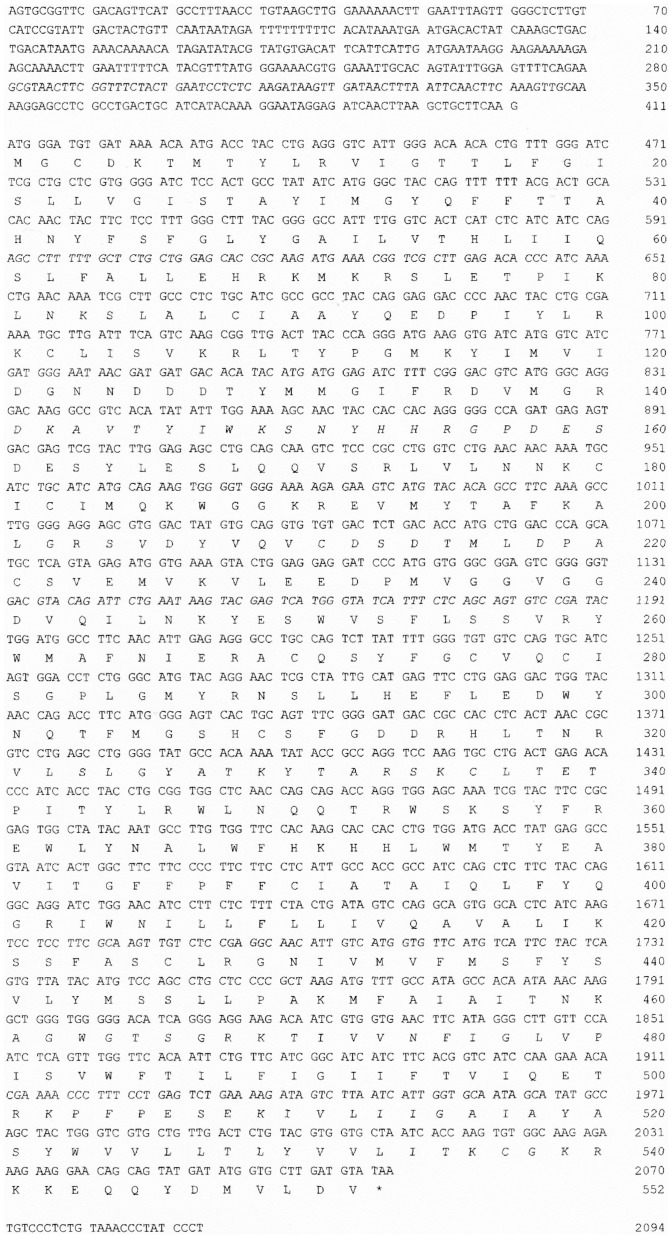
Nucleotide sequence of Japanese eel hyaluronan synthase 2 (eHAS2) with the predicted amino acid sequence given below.

**Figure 3:**
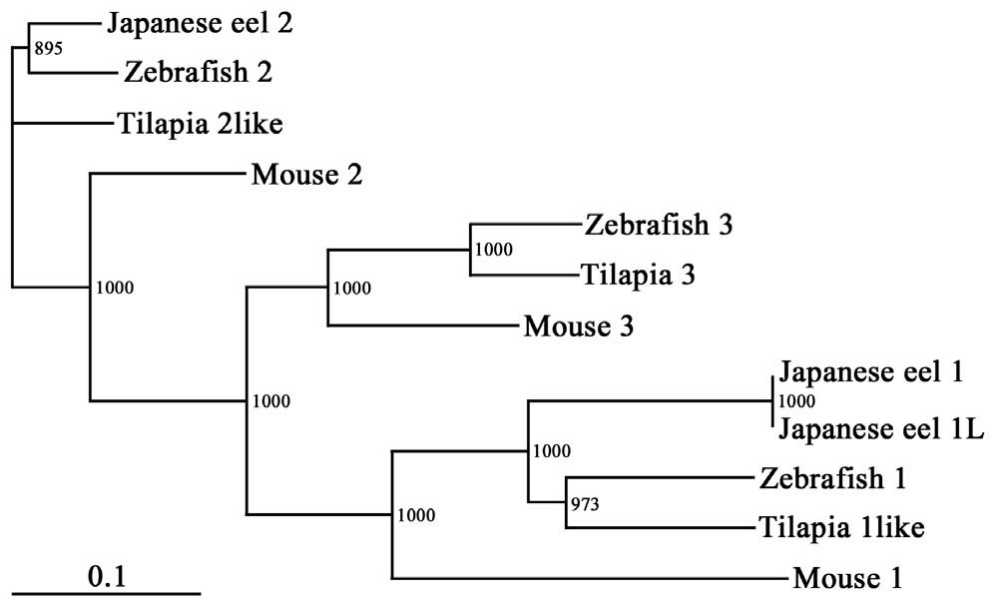
Phylogenetic tree of 12 vertebrate hyaluronan synthases (HASs). The horizontal lines indicate genetic distance. One thousand bootstrap replicates were performed; values are shown at the inner nodes.

**Figure 4:**
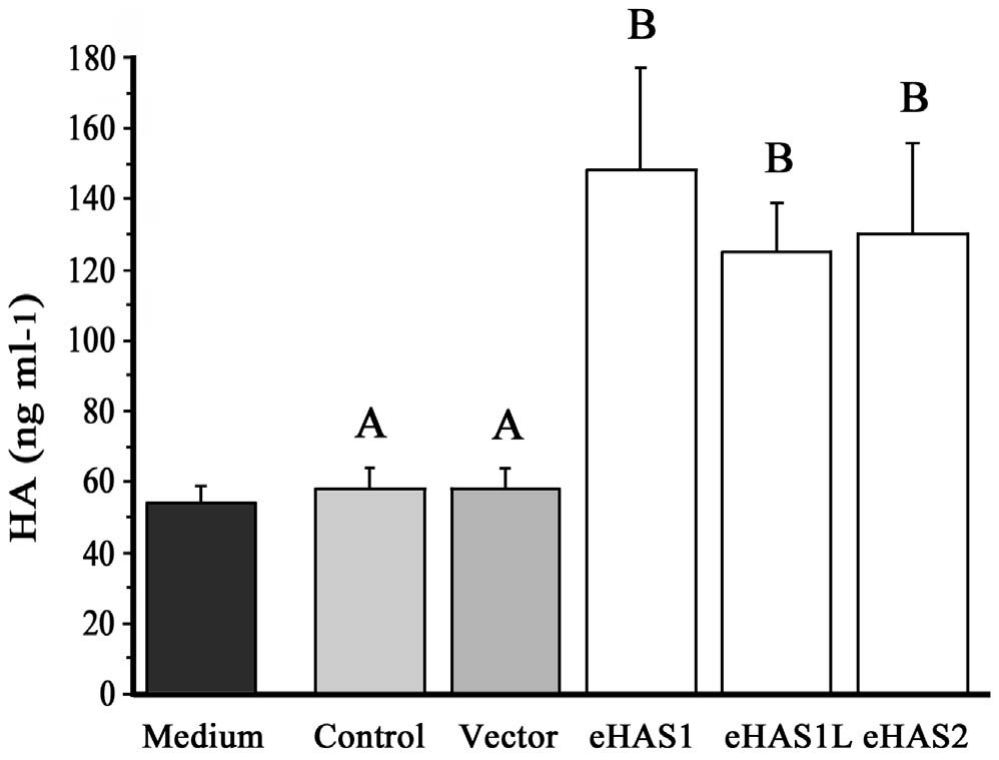
HA synthesis activity of transfected eHAS1 and eHAS2 for Hepa-E1 cells. Hepa-E1 cells were transfected with the pcDNA-eHAS1 or pcDNA-eHAS2 vectors. Cells were seeded at a density of 0.4×10^5^ per well and incubated for 48 h. Each value represents the mean ± SEM of four independent experiments. The mean HA value of the control (not transfection expression vector) was set at a relative of 1. Medium: HA contents in E-RDF medium supplemented with 5% FBS. Control: HA contents in the medium using a cell culture. Vector: HA contents in the medium using a cell culture transfected with the pcDNA3.1(+). eHAS: HA contents in the medium using a cell culture transfected with the pcDNA-eHAS. Mean values with the same eHAS expression vector and sharing the same letter label did not differ significantly (Tukey-Kramer HSD test, *p*<0.05).

HA is synthesized by integral plasma membrane glycosyltransferases and is exported directly into the extracellular space. Three distinct yet highly conserved genes encoding HAS, HAS1 [24]–[26], HAS2 [27], [28], and HAS3 [29] were cloned. The three gene products are similar in amino acid sequence and molecular structural characteristics. In mammals, three HASs synthesize HA; however, HAS activity differs between the three [30]. The eHAS1 and eHAS2 nucleotide and deduced amino acid sequences are shown in Figures 1 and 2. The cDNA encoding eHAS1 contains a complete putative open reading frame of 1,701 bp, which encodes a putative protein of 567 amino acid residues. Another type of eHAS1, a splice variant named eHAS1L, has a 35 amino acid insertion. The cDNA encoding eHAS2 contains an open reading frame of 1,656 bp, encoding 552 amino acid residues. When the amino acid sequence corresponding to the Japanese eel genes were compared with that of other known HAS genes, the proteins exhibited the highest homology to teleost HAS1 and HAS2 (Figure 3). Moreover, eHAS1 and eHAS2 induce HA synthesis (Figure 4).

**Figure 5:**
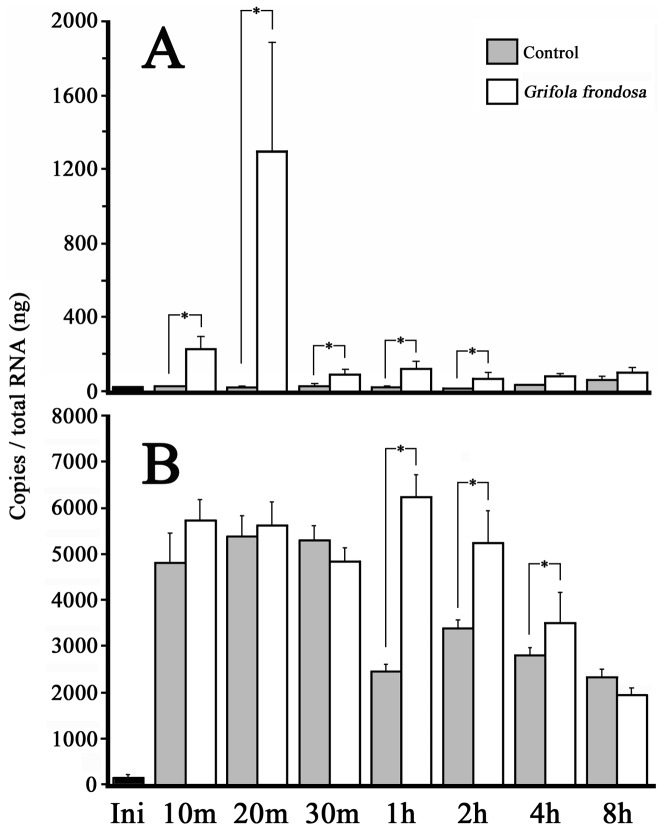
Changes in larval (A) eHAS1s and (B) eHAS2 mRNA expression levels (7 dph) after being fed a slurry-type diet with *Grifola frondosa* extract. Initial: 7 dph fish with no feed. 10 min to 8 h: the time span over which the 7 dph fish were fed. Control: a normal slurry-type diet, *G. frondosa*: a slurry-type diet with *G. frondosa* extract. Values represent the means ± SEM of six independent pooled samples. *indicates significant differences between the control and *G. frondosa* the same amount of time after feeding (Mann-Whitney *U*-test, *p*<0.05).

**Figure 6:**
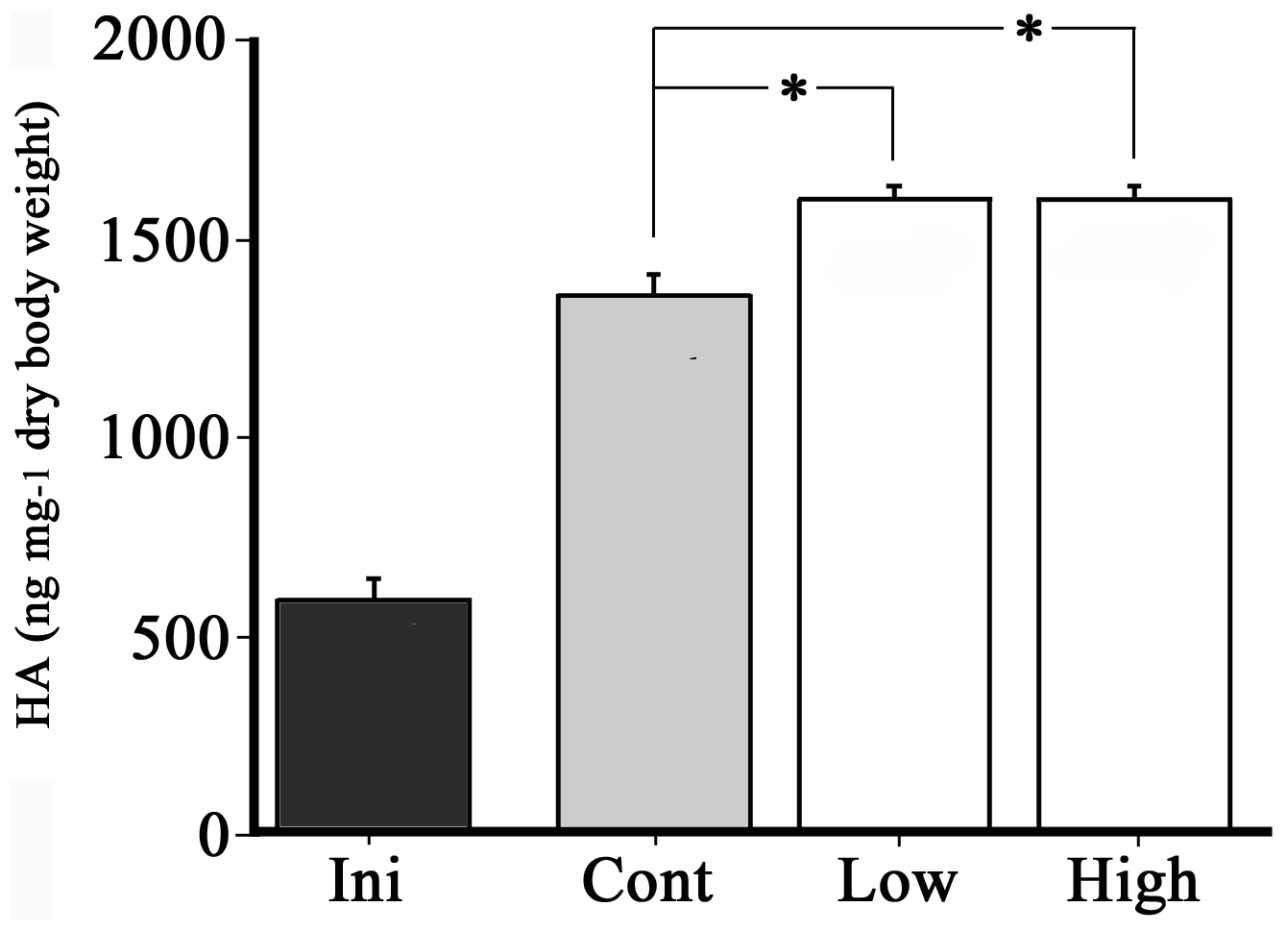
Effect of *Grifola frondosa* extract on HA content at 27 dph. Cont: control diet (a normal slurry-type diet), Low: 0.2 mg/g *G. frondosa* extract in a slurry-type diet, High: 2 mg/g *G. frondosa* extract in a slurry-type diet. Each value represents the mean ± SEM of four independent experiments. *indicates significant differences between the Control, Low, and High diets (Tukey-Kramer HSD test, p<0.0001).

Figure 5 shows the changes in eHAS1 and eHAS2 mRNA expression levels after being fed a diet both with and without *Grifola frondosa* extract, which is a HA synthesis-promoting substance (HASPS). Initially, when first-feeding larvae did not feed, eHAS1 and eHAS2 mRNA levels were low. As for eHAS1s, they remained the same after the eels were fed the control diet. eHAS1 mRNA expression was elevated after they were fed the *G. frondosa* extract diet, and peaked after 20 min. Between 10 min and 2 h eHAS1 mRNA expression was higher in eels fed the *G. frondosa* extract diet than in those fed the control diet. In contrast, eHAS2 was elevated after they were fed the control diet, continued to increase for 30 min, and subsequently decreased after 1 h. In teleosts, the relationship between HAS synthesis and feeding is unknown; however, from this result, it seems possible that feeding activity and/or this diet with added maltose activates eHAS2 expression. In contrast, there was no significant difference in eHAS2 mRNA expression between the control diet and *G. frondosa* extract diets after 10, 20, or 30 min. After 1, 2, and 4 h eHAS2 mRNA expression was higher in eels fed the *G. frondosa* extract diet than in those fed the control diet. The addition of *G. frondosa* extract to human skin fibroblast cells *in vitro* activates HAS2 mRNA expression and secretes increasing quantities of HA [15]. In this experimental model, if we fed the *G. frondosa* extract diet to first-feeding larvae, eHAS1 (containing eHAS1L) mRNA expression was higher than in the control diet for 2 h and high eHAS2 mRNA expression continued longer than in the control diet (Figure 5). Moreover, larval HA content was significantly increased by *G. frondosa* extract (Figure 6). This is the first report in non-mammal vertebrates that HASPS increase the amount of HA in the body. Based on our results, we speculate that the long-range activity of eHASs, at least eHAS1 and eHAS2, promote HA synthesis.

At present, the exact substance, including HASPS extracted from *G. frondosa* or seaweeds, that stimulates HA production is unknown. The existence of glycerophospholipids in *G. frondosa* has been reported previously [31]. The addition of phosphatidylserine and/or phosphatidylinositol to human fibroblast cells *in vitro* significantly increases HAS2 mRNA expression and HA content [32]. It is highly possible that glycerophospholipid activates HA synthesis in Japanese eel larvae. However, this requires further work as the exact mechanism is unknown.

**Figure 7:**
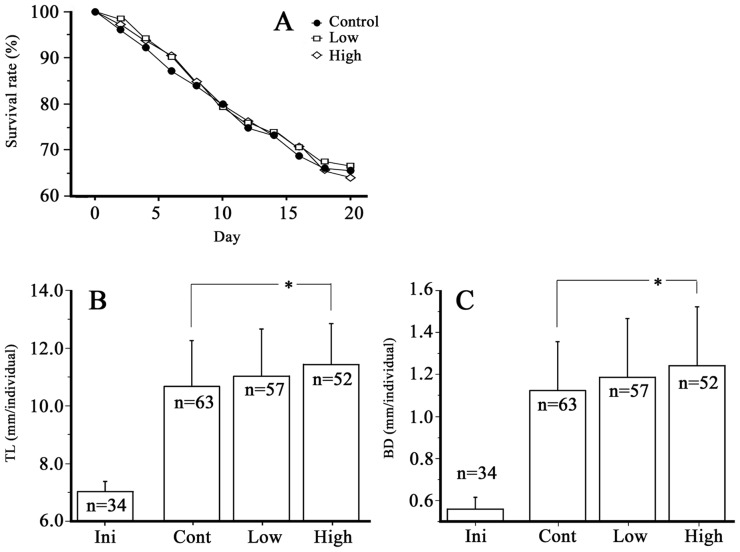
Effects of Grifola frondosa extract on survival rate (A), total length (TL) (B), and body depth (BD) (C) at 27 dph. Cont: control diet (a normal slurry-type diet), Low: 0.2 mg/g *G. frondosa* extract in a slurry-type diet, High: 2 mg/g *G. frondosa* extract in a slurry-type diet. Each value of B and C represents the mean ± SDM. *indicates significant differences between the Control, Low, and High diets (Tukey-Kramer HSD test, B: p<0.05, C: p<0.01).

**Figure 8:**
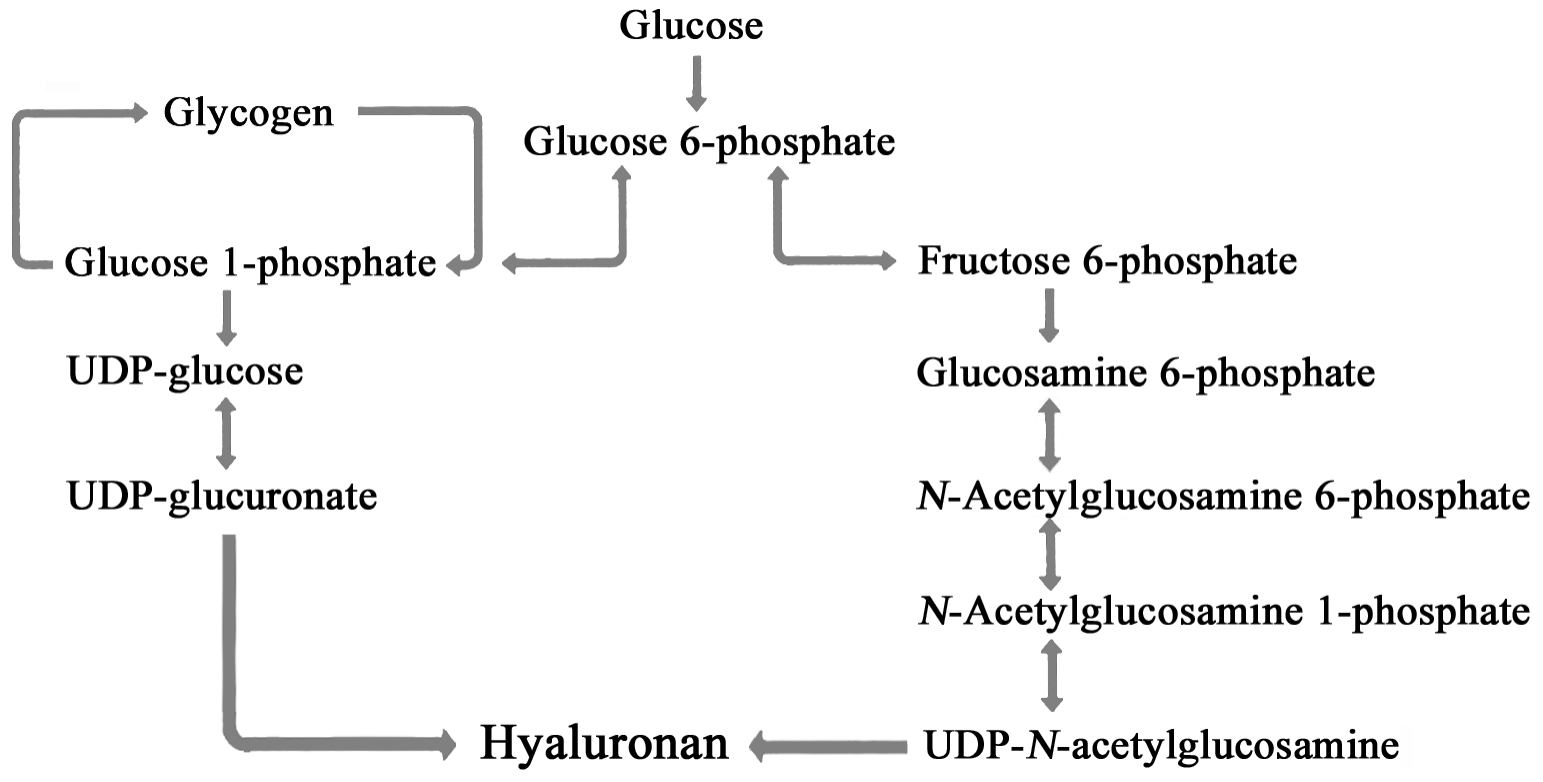
Hyaluronan pathway.

Figures 7 shows the survival rate and growth (TL and BD) results from the larvae fed the HASPS derived from *G. frondosa* extract. As for survival rates, we did not see a significant difference between the control and the HASPS. However, TL and BD in the HASPS experiment exhibited a significant increase compared with those of the control [*G. frondosa* extract (2 mg/g) vs. control]. A previous study, using bonefish (*Albula* sp.) leptocephali, looked at energy budgets during metamorphosis, part of the energy was provided by GAGs [14] and it was suggested that HA is a major energy source [12]. The relationships between accumulated HA and growth in vertebrates is unknown. In this study, HASPS enhanced leptocephalus growth; however, it is not clear whether or not accumulated HA in the body directly enhanced leptocephalus growth. Figure 8 shows the interrelationships in HA metabolism [33]. HA is synthesized in UDP-GlcNAc and UDP-GlcUA by HAS [33], moreover, glucose is the precursor of UDP-GlcNAc and UDP-GlcUA. We hypothesize that a reversible relationship exists between neutral sugars and HA. In other words, in leptocephali, HA stored energy sources are metabolized to sugars when metabolic energy is needed. In our style of seeding culture for Japanese eel, it is difficult for leptocephali to feed continuously. Because the slurry-type diet causes deterioration of the culture water [1], we must wash away the food immediately after feeding. Assuming that HA is a source of stored energy, this style of feeding may be advantageous for larval growth. In other words, feeding style might have caused the differences in leptocephalus growth whether or not HASPS were added.

## Materials and Methods

### Ethics

The fish handling, husbandry, and sampling methods were approved by the Institutional Animal Care and Use Committee of National Research Institute of Aquaculture (IACUC-NRIA No. 25008).

### *Grifola frondosa* extract

Commercially-supplied *Grifola frondosa* fruit bodies were prepared and left to dry naturally. When dried, *G. frondosa* was mixed with 10 times its mass of 100% ethanol and shaken overnight at room temperature (RT). The extracted ethanol was filtered with filter paper and freeze-dried in a freeze dryer.

### Japanese eel larvae

Cultivated adult male Japanese eels (150–200 g body mass) were purchased from a commercial supplier. As for the adult female supply, glass-eels from a commercial eel supplier were feminized by feeding them estradiol-17β (Sigma, St. Louis, MO). The fish were kept at the Nansei Station, National Research Institute of Aquaculture, Fisheries Research Agency. Artificial maturation was carried out by hormone treatment as previously described [17], [18]. Females were repeatedly injected with salmon pituitary extract, followed by injection with 17α-hydroxyprogesterone (Sigma). Similarly, males purchased from a commercial supplier were injected with human chorionic gonadotropin (ASKA Pharmaceutical Co. Ltd., Tokyo, Japan). The above hormone treatments were performed according to Kagawa et al. [19]. The gametes were obtained by gently stripping ovulating females and mature males.

Larvae hatched fertilized eggs were maintained in an acrylic tank at 25°C with running seawater. After the first-feeding [7 or 8 days post hatching (dph)], a slurry-type diet consisting of shark eggs, soybean peptides (Fuji Oil Co. Ltd., Osaka, Japan), krill hydrolysate (Nippon Suisan Kaisha, Ltd., Tokyo, Japan) and krill extract [4], [6] was given to the larvae 5 times a day at 2-h intervals from 9 am to 5 pm. Fully-grown leptocephali were sampled, frozen in liquid nitrogen, and stored at −80°C until required.

### Reverse transcription (RT)-PCR and cDNA cloning of Japanese eel hyaluronan synthase 1 and 2

Total RNA was extracted from fully-grown leptocephali using Isogen (Nippon Gene, Tokyo, Japan). Poly (A) + RNA was subsequently isolated from total RNA using Oligotex-dt-30 (Takara, Otsu, Japan). Isolated RNA was denatured at 70°C for 10 min, placed on ice, and reverse transcribed with M-MLV. Second-strand cDNA was synthesized and single-strand overhands were removed, using Takara's cDNA cloning system (Takara).

Japanese eel hyaluronan synthase 1 (eHAS1) and 2 (eHAS2) cDNA fragments were amplified using sense and antisense degenerate primers designed based on a consensus sequence from the aligned deduced amino acid sequences of HAS from several vertebrate species (Table 1). PCR was carried out in a final volume of 50 µl containing 0.5–1 pg cDNA, 400 nM of each primer, 800 µM of each dNTP, and 2.5 U Ex Taq (Takara). PCR was carried out for 35 cycles in a Thermal Cycler Dice Gradient (Takara) under the following conditions: denaturation at 94°C for 30 s, annealing at 50–55°C for 30 s, and extension at 72°C for 20–30 s. PCR products were separated by 1% agarose gel electrophoresis, and selected bands were cut out and purified with a QiAprep Spin Miniprep Kit (Qiagen, Venlo, the Netherlands). Purified DNA fragments were subcloned into the plasmid vector pGEM-T Easy (Promega, Madison, WI, USA), and positive clones were sequenced with a Big Dye Terminator v3.1 Cycle Sequencing Kit (Applied Biosystems, Foster City, CA, USA) and an ABI PRISM 3100 Genetic Analyzer (Applied Biosystems).

**Table 1:** Primers used for cloning, PCR, and real-time RT-PCR analysis of Japanese eel HAS1 (eHAS1) and HAS2 (eHAS2).

Name	Primers sequences (5′-3′)	Nucleotide numbers corresponding to the annealing site
HAS1-DP-S	TGGGGNGGNAARMGNGARGTGATG	
HAS1-DP-A	AKCGSGTYTGYTGRYTSAGCC	
HAS2-DP-S	TAYTGGATGGCKTTYAAYATWGA	
HAS2-DP-A	ATSACNGCYTCRTAGGTCATCCA	
		
eHAS1-UTR-S	GTTCCACTTGTATATAAGTATTCTGCC	1–27 bp (Fig. 1)
eHAS1-UTR-A	GTGAAACAGTGTATTATTTGTATTTTGC	2668–2695 bp (Fig. 1)
eHAS2-UTR-S	AGTGCGGTTCGACAGTTCATGC	1–22 bp (Fig. 2)
eHAS2-UTR-A	AGGGATAGGGTTTACAGAGGG	2074–2094 bp (Fig. 2)
		
eHAS1-5RACE-A1	ACTTTGTCCAGCGCGTCTGCTGGTT	1349–1373 bp (Fig. 1)
eHAS1-5RACE-A2	GCGGGTCCAGCTTGGTATCTGAG	940–962 bp (Fig. 1)
eHAS1-3RACE-S1	TGTGGAACGAGCCTGCCAGTCCT	1099–1121 bp (Fig. 1)
eHAS1-3RACE-S2	TGTGTCTCCTGCATCAGTGGCCC	1130–1152 bp (Fig. 1)
eHAS2-5RACE-A1	ATTTGCTCCACCTGGTCTGCTGGTT	1456–1480 bp (Fig. 2)
eHAS2-5RACE-A2	ATTTTGTGGCATACCCCAGGCTCAG	1375–1399 bp (Fig. 2)
eHAS2-3RACE-S1	TGCCAGTCTTATTTTGGGTGTGTCCAGT	1219–1246 bp (Fig. 2)
eHAS2-3RACE-S2	TCAACCAGCAGACCAGGTGGAGC	1454–1476 bp (Fig. 2)
		
eHAS1-ST-S	GGCTGATCATGACGCAGATAAAAATGTCGTAACAACTCCG	
eHAS1-ST-A	ACACATTGACGCCTGCATTAACCAAGCTTAAGTTTAAACG	
eHAS1-S	GGCTGATCATGACGCAGATA	2486–2505 bp (Fig. 1)
eHAS1-A	ACACATTGACGCCTGCATTA	2627–2646 bp (Fig. 1)
eHAS2-ST-S	TGGGCTCTTGTCATCCGTAAAAATGTCGTAACAACTCCG	
eHAS2-ST-A	TGTGCAATTTCCACGTTTTCACCAAGCTTAAGTTTAAACG	
eHAS2-S	TGGGCTCTTGTCATCCGTA	60–78 bp (Fig. 2)
eHAS2-A	TGTGCAATTTCCACGTTTTC	242–261 bp (Fig. 2)
		
pcDNA-eHAS1-S	TTTAAACTTAAGCTTGCGCAATAAA ATACCGGTCT A	
pcDNA-eHAS1-A	TGGACTAGTGGATCCACGCTGTAACTTTCTGACCGT	
pcDNA-eHAS2-S	TTTAAACTTAAGCTTATCAACTTAAGCTGCTTCAAG	
pcDNA-eHAS2-A	TGGACTAGTGGATCCGATAGGGTTTACAGAGGGACA	

DPs (degenerate primers), primers for amplification of eHAS fragments; eHAS-UTRs (untranslated regions), sense and antisense primers for the sequencing of eHAS containing the open reading frame; eHAS-3RACE, eHAS-5RACE, sense, and antisense primers for the sequencing of 3′- and/or 5′-RACE analysis of eHAS. eHAS-ST (standard), sense, and antisense primers standard real-time RT-PCR analysis of eHAS; eHAS, sense, and antisense primers for real-time RT-PCR analysis of eHAS; pcDNA-eHAS, sense, and antisense primers for construction of eHAS expression when inserted into the pcDNA3.1(+) vector.

### 5′ and 3′ rapid amplification of cDNA ends (RACE)-PCR

Fully-grown leptocephali were used for the construction of cDNA for RACE-PCR with a SMART RACE cDNA Amplification Kit (Clontech, Palo Alto, CA). For both 3′-RACE and the 5′-RACE, nested primers (eHAS-3RACE-S1, -S2 and eHAS-5RACE-A1 and -A2, respectively) were designed from eHAS cDNA fragments (Table 1). Based on the eHAS cDNA fragments amplified by 5′- and 3′-RACE-PCR, sense (eHAS-UTR-S) and antisense (eHAS-UTR-A) primers were designed for the untranslated regions (Table 1). PCR was carried out as described above.

These PCR products were sequenced following the method described above, and eHAS cDNA sequences containing the entire open reading frame were obtained. eHAS cDNA products representing independent, full-length PCR clones were each sequenced 5 times to detect PCR errors.

### Sequence analysis

The GenBank accession numbers of the sequences compared with our eHAS1s and eHAS2 sequence were: eHAS1, eHAS1L, and eHAS2 (AB901107, AB901106, and AB823817), zebrafish (*Danio rerio*) HAS1, HAS2, and HAS3 (NM_001164030, AF190742, and AF190743), Nile Tilapia (*Oreochromis niloticus*) HAS1-like, HAS2-like, and HAS3 (XM_003458416, XM_003443695, and XM_003449190), mouse (*M. musculus*) HAS1, HAS2, and HAS3 (NM_008215, NM_008216, and NM_008217).

A phylogenetic tree was constructed using the neighbor-joining method [20]. For this analysis, 1,000 bootstrap replicates were carried out using ClustalW version 2.1 (DNA Data Bank of Japan, http://www.ddbj.nig.ac.jp/index-j.html).

### Construction and transfection of eHAS1 and eHAS2 expression vectors

Single-stranded cDNA was prepared from the poly(A)+ -RNA of Japanese eel tissue by the method described above. The entire eHAS coding regions were amplified by PCR using primers that introduced a 15 bp (5′-TTTAAACTTAAGCTT-3′) pcDNA3.1 (+) (Invitrogen) sequence at the 5′ end (pcDNA-eHAS-S), a 15 bp sequence (5′-TGGACTAGTGGATCC-3′) at the 3′ end (pcDNA-eHAS-A), and Ex-Taq (Takara) (Table 1). The eHAS fragments were inserted into pcDNA3.1(+), which contains the cytomegalovirus (CMV) promoter upstream of the inserted cDNAs (pcDNA-eHAS). The BamHI and HindIII sites were digested with an In-Fusion HD cloning Kit (Takara). The cDNA products were sequenced and we confirmed that there were no errors arising from the PCR.

Hepa-E1 cells, epithelial-like Japanese eel hepatocytes that have no TH deiodinase activity [21], were obtained from the Institute of Physical and Chemical Research (RIKEN) cell bank (Tsukuba, Japan). Hepa-E1 cells were seeded in 48-well plates at densities of 0.4×10^5^ cells/well in an E-RDF medium (Kyokuto, Tokyo, Japan) supplemented with 5% fetal bovine serum (FBS) (Sigma). After a further 24 h of incubation, the cells were transfected with 300 ng pcDNA-eHAS using X-treme GENE 9 DNA Transfection Reagent (Roche, Mannheim, Germany). The transfected Hepa-E1 cells were cultured for 1 day in the above medium with FBS containing 1 mM Uridine diphosphate (UDP)-GlcUA (Nacalai Tesque, Kyoto, Japan) and 1 mM UDP-GlcNAc (Sigma) at 28°C.

### eHAS synthesized HA

HA synthesized by the eHAS transfectant was analyzed according to Kawakami et al. [13]. After 2 days of transfection, the removed medium was mixed with 20 µg/µl actinase E (Kaken Pharmaceutical, Tokyo, Japan), and incubated at 50°C for 24 h. The mixture was boiled for 10 min and then centrifuged at 5,000×g for 10 min. The supernatant was then used for HA content measurement using an assay kit (Funakoshi, Tokyo, Japan). The intra-assay coefficients of variation were 0.0–5.0%.

### Experiment 1: Culture experiment: eHAS analysis of first-feeding larvae fed a *G. frondosa* extraction diet

The first-feeding larvae (7 dph) were fed the slurry-type diet, which consisted of 200 mg of *G. frondosa* extract and 2 g maltose (Wako, Tokyo, Japan) diluted in a measuring cylinder to 10 mL with 0.8% xanthan gum (Wako). For the control, 2 g maltose (Wako) diluted in a measuring cylinder to 10 mL with 0.4% xanthan gum (Wako) was supplied.

About 2,000 larvae at 7 dph, the duration of first feeding, were moved to a 10-L acrylic tank and fed the slurry-type diet on the bottom of the tank as described by Tanaka et al. [1]. After 15 min the feed was washed out. Subsequently, approximately 100–200 larvae were sampled after 10, 20, 30 min, 1, 2, 4, and 8 h into RNAlater (Ambion, Austin, TX, USA) and stored at −20°C until required.

### Experiment 1: Real-time RT-PCR: eHAS analysis of first-feeding larvae fed the *G. frondosa* extraction diet

Real-time RT-PCR analyses were performed using a MyiQ Real-Time Detection System (Bio-Rad, Hercules, CA, USA). Specific primers for eHAS1 and eHAS2 were designed based on the cDNA nucleotide sequence analysis in this study (Table 1). As the primers designed for eHAS1 amplify both eHAS1 and a splice variant of eHAS1 (eHAS1L), the real-time RT-PCR method estimates the relative abundance of mRNA for both eHAS1s.

Total RNA from 30 to 50 embryos/fish, was extracted according to Kawakami et al. [22], [23]. Synthesis of first-strand cDNA was carried out as follows: 100 ng of isolated total RNA was reverse-transcribed with a SuperScript VILO cDNA synthesis Kit (Invitrogen, Carlsbad, CA, USA).

As a standard, appropriate sizes of pcDNA3.1(+) (Invitrogen) fragments flanked by primer-binding sites were prepared by PCR as above. PCR fragments for the standard were discriminated by gel electrophoresis and purified with a QIAquick Gel Extraction kit (Qiagen, Hilden, Germany). Purified PCR fragments were quantified by UV260 absorbance and a 1/100 dilution series was constructed (10^9^–10^1^ copies/µl). RT-PCRs and a dilution series of standard samples were prepared according to the manufacturer's protocol to include: 1 ng of cDNA, 5 µl of SsoAdvanced SYBR Green Supermix (Bio-Rad), and 500 nM primers in a final volume of 10 µl. DNA amplifications were performed in duplicate (standards: triplicate) under the following conditions: 30 s at 95°C, followed by 40 cycles of 5 s of denaturation at 95°C, 10 s of annealing, and extension at 60°C for 20 s. Standard templates were used, in triplicate, to construct a standard curve ranging from 10^1^ to 10^9^ copies. The linear range of the curve fell within 10^1^–10^9^ copies and the correlation coefficients of variation were greater than 0.997 for all curves. The intra-assay coefficients of variation were 4.8–22.2%. Inter-assay coefficients of variation were under 10.0% for analysis.

### Experiment 2: Culture experiment: Effects of *G. frondosa* extract on larval growth and HA content

For the culture experiment using first-feeding larvae (7 dph), we prepared a slurry-type diet, as above, adding *G. frondosa* extract (0.2 or 2 mg/g). First-feeding larvae (7 dph), 200 per tank, were moved to a 5-L acrylic tank at 25°C with running seawater. The above mentioned slurry-type diet was given to the larvae 5 times a day at 2-h intervals from 9 am to 5 pm. Each experiment was duplicated and feeding continued for 20 days (7 to 26 dph). After the culture experiment, the total larval length (TL) and body depth (BD) were measured, about 20 to 30 larvae per a lot were sampled, frozen in liquid nitrogen, and stored at −80°C until required.

### Experiment 2: HA analysis: Effects of *G. frondosa* extract on larval growth and HA content

A sample of 20 to 30 larvae was treated according to Kawakami et al. [13]. Treated samples (in duplicate) were then used for HA content measurement using an assay kit (Funakoshi). The intra-assay coefficients of variation were 0.4–5.5%.

### Data analysis

eHAS1s and eHAS2 expression data were arcsine transformed (√%). The difference in HA synthesis activity of transfected eHAS1 and eHAS2 for Hepa-E1 cells and the effect of *Grifola frondosa* extract on HA content, total length, and body depth at 27 dph were analyzed by one-way ANOVA and the Tukey-Kramer honestly significant difference (HSD) *post hoc* test for individual analyses. eHAS mRNA expression levels after being fed a slurry-type diet with *Grifola frondosa* extract was subjected to Mann-Whitney U tests.
